# Measurement of β-isomerized C-terminal telopeptide of type I collagen in patients with POEMS syndrome: diagnostic, prognostic, and follow-up utilities

**DOI:** 10.1038/bcj.2016.109

**Published:** 2016-11-11

**Authors:** X Huang, C Zhang, C Wang, Q Cai, X Cao, H Cai, L Zhang, J Feng, D Zhou, J Li

**Affiliations:** 1 Department of Hematology, Peking Union Medical College Hospital, Chinese Academy of Medical Sciences and Peking Union Medical College, Beijing, China

POEMS (polyneuropathy, organomegaly, endocrinopathy, monoclonal gammopathy and skin changes) syndrome is a paraneoplastic disorder, of which most clinical manifestations are attributed to elevated vascular endothelial growth factor (VEGF). Osteosclerosis, although well described in the majority of patients, has an obscure mechanism. Although conventional imaging methods, including plain radiography and computed tomography, can be informative, these techniques are neither sensitive nor specific enough to detect the sclerotic changes.^1, 2^ Markers of bone turnover are easily measured, and when interpreted in the context of appropriate clinical design can provide valuable information. Indeed, increased bone turnover markers were reported in POEMS patients, and have even been suggested for inclusion into the diagnostic criteria.^3, 4^

Carboxy-terminal telopeptide (CTX) is formed during type I collagen degradation in the bone, and an isomerization reaction occurs subsequently under physiological conditions. The resulting β-isomerized CTX (β-CTX) can be measured automatically in most clinical laboratories, and is used to assess bone diseases.^5, 6^ Except for the aforementioned studies concerning the diagnostic performance of bone turnover markers in POEMS syndrome, there is nearly no information about their levels in response to effective treatment. Moreover, their potential utility in therapeutic evaluation and follow-up is also poorly understood.

The current study included 146 POEMS patients who were diagnosed and treated at Peking Union Medical College Hospital (Beijing, China) between January 2011 and March 2016. All met the diagnostic criteria proposed by Dispenzieri.^2^ Clinical and laboratory information was collected, as described previously.^1, 7^ Serum β-CTX and N-terminal propeptide of type I collagen (PINP) levels were measured using an automatic analyzer (Roche Cobas E601; Holliston, MA, USA) with Elecsys reagent kits (Roche Diagnostics, Basel, Switzerland). Serum VEGF was measured with a human Quantikine ELISA Kit (normal <600 pg/ml; R&D Systems, Minneapolis, MN, USA).^3^ Detailed methods are provided in the Online Supplementary Materials.

The patients' characteristics are shown in Table 1. In the beginning of our study, we measured both serum β-CTX and P1NP in 43 POEMS patients and observed a statistically significant linear correlation between these two markers (Spearman ρ=0.341, *P*=0.025; Online Supplementary Figure 1), indicating that bone formation and resorption processes are well coupled in these patients. Considering both the assay stability and patients' financial reasons, we chose to measure β-CTX alone in the following study.^8^ There is no significant difference in clinical characteristics between patients with (*n*=43) and without PINP (*n*=103), indicating a good representativeness of the early cohort.

The median level of serum β-CTX was 1.000 ng/ml (range, 0.300–3.500 pg/ml), with median values of 1.050 ng/ml (range, 0.300–3.500 pg/ml) and 0.950 ng/ml (range, 0.330–2.440 ng/ml) in males and females, respectively, (*P*=0.286). One hundred and thirty-five subjects (92.5% 83 of 92 in males (90.2%); 52 of 54 in females (96.3%)) had elevated levels at diagnosis. A statistically significant correlation was observed between the serum levels of β-CTX and VEGF (Spearman ρ=0.198, *P*=0.021; Figure 1a).

Serum levels of β-CTX were markedly higher in POEMS patients compared with normal subjects (median, 0.300 ng/ml; *P*<0.001), patients with chronic inflammatory demyelinating polyneuropathy (median, 0.400 ng/ml; *P*=0.014), systemic lupus erythematosus (median, 0.700 ng/ml; *P*=0.001) or Langerhans cell histiocytosis (median, 0.343 ng/ml; *P*<0.001), but not multiple myeloma (median, 0.946 ng/ml; *P*=0.602; Figure 1b). Using a receiver operating characteristic analysis, the best β-CTX cutoff value for diagnosing POEMS was 0.576 ng/ml, with a specificity of 63.7% and a sensitivity of 90.4%. The area under the curve was 0.81 (95% confidence interval 0.75–0.87, *P*<0.001). When we designated 1.000 ng/ml as the cutoff value, the specificity improved to 82.2%, with a decline in sensitivity (47.3%).

When dividing patients into two subgroups according to the median levels in each gender, we found high β-CTX levels at baseline were associated with several POEMS manifestations (Table 1). As serum β-CTX is a bone turnover marker, we also compared its levels in patients with (*n*=98) and without (*n*=48) osteosclerosis. No statistical difference was found (median, 1.030 vs 1.000 ng/ml, *P*=0.473).

After primary therapy, serum β-CTX levels were significantly reduced (median, 0.600 ng/ml; range, 0.090–2.200 ng/ml, *P*<0.001), and 51 of 135 patients (37.8%) achieved normalization. In terms of other responses, 78 of 129 (60.5%) and 53 of 137 patients (38.7%) had VEGF and hematological remissions, respectively. Moreover, normalization of β-CTX was strongly linked to VEGF (*P*=0.027) and hematologic remissions (*P*=0.006).

With a median follow up of 24.2 months, five patients died and 14 had disease progression. The estimated 3-year overall survival (OS) and progression-free survival (PFS) were 96.4% and 80.5%, respectively. Patients with β-CTX normalization after treatment had a superior 3-year OS (100% vs 93.4%, *P*=0.043) and a lower risk of progression (11.1% vs 29.1%, *P*=0.025) (Figure 1c). Similar favorable outcomes were observed in patients with VEGF (3-year OS, 100% vs 89.6%, *P*=0.022; 3-year progression risk, 12.2% vs 43.9%, *P*=0.001) and hematologic remissions (3-year OS, 100% vs 93.7%, *P*=0.057; 3-year progression risk, 8.9% vs 31.9%, *P*=0.011) (Online Supplementary Figure 2). Notably, serial measurements of β-CTX and VEGF in several patients with a relapsed disease course showed that β-CTX levels could still be abnormal, even in patients who achieved VEGF normalization (Online Supplementary Table).

We have demonstrated that serum β-CTX levels are elevated in POEMS patients and correlate with the well-characterized disease marker, VEGF. The two have similar diagnostic accuracies. However, β-CTX is routinely measured in most hospitals, whereas the VEGF assay is typically performed in reference laboratories only, and results take several days or even longer. The availability and relatively short turnaround time may help spread the clinical use of β-CTX. Furthermore, we have shown the utility of serum β-CTX in disease monitoring, and its normalization after therapy could predict superior OS and less relapse. It is noteworthy that β-CTX levels could still be abnormal, even in patients who achieved VEGF normalization, suggesting that disease activity in POEMS syndrome is multifaceted. Responses in different categories, although always strongly associated, do not completely overlap, and have separate roles in therapeutic evaluation. These findings may prompt the clinical usage of this easily measured bone turnover marker in POEMS syndrome management.

## Figures and Tables

**Figure 1:**
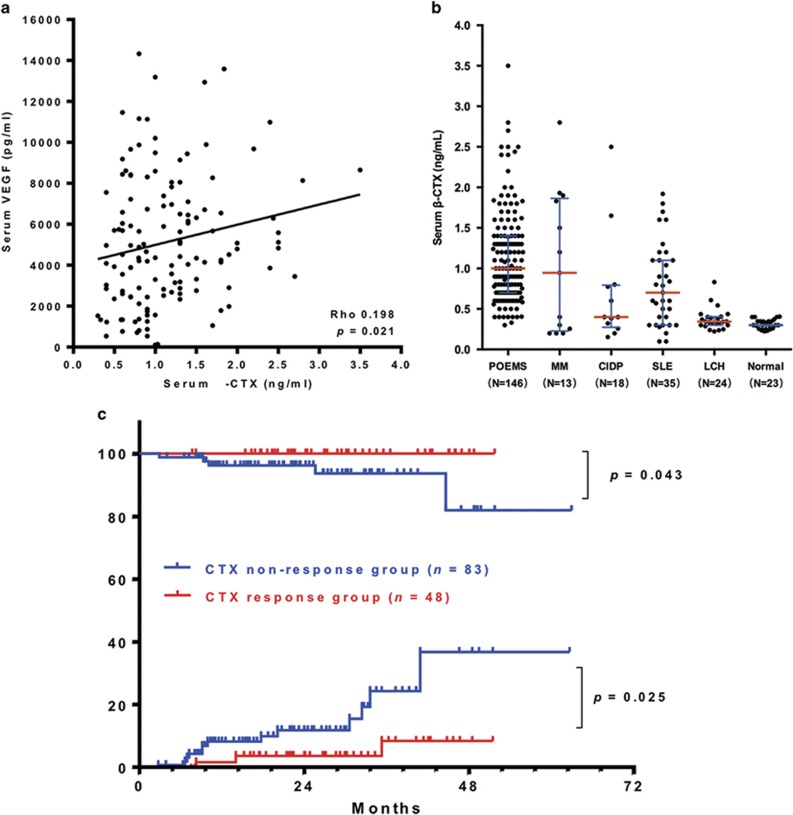
(**a**) Correlation between serum levels of β-CTX and VEGF in POEMS patients. (**b**) Distribution of serum levels of β-CTX in POEMS patients and controls. Error bar: interquartile range. (**c**) Overall survival and risk of progression in POEMS patients with and without β-CTX normalization therapies.

**Table 1:** Patient characteristics

*Characteristics*	*All (*N*=146)*	*Higher β-CTX (*N*=73)*	*Lower β-CTX (*N*=73)*	P-*values*^a^
Age (median, range)	48 (21–74)	47 (21–74)	48 (25–65)	0.124
Male (*N*, %)	92 (63.0%)	46 (63.0%)	46 (63.0%)	1.000
				
*Polyneuropathy*				
ONLS score (median, range)	4 (1–11)	4 (1–11)	3 (1–10)	0.036
				
*Organomegaly*				
Lymphadenopathy	91 (62.3%)	52 (71.2%)	39 (53.4%)	0.026
Splenomegaly	88 (60.3%)	45 (61.5%)	43 (58.9%)	0.735
Hepatomegaly	68 (46.6%)	37 (50.7%)	31 (42.5%)	0.320
				
*Endocrinopathy*				
Diabetes mellitus	20 (13.7%)	11 (15.1%)	9 (12.3%)	0.630
Hypothyroidism	96 (65.8%)	54 (74.0%)	42 (57.5%)	0.036
				
*Monoclonal gammopathy*				
M-protein (g/l) (median, range)	0.0 (0.0–13.5)	0.0 (0.0–13.5)	0.0 (0.0–9.0)	0.574
Heavy chain use: IgA	93 (63.7%)	48 (65.8%)	45 (61.6%)	0.606
				
*Skin changes*				
Hyperpigmentation	129 (88.4%)	67 (91.8%)	62 (84.9%)	0.197
Hemangioma	94 (64.4%)	48 (65.8%)	46 (60.3%)	0.730
Castleman's disease^b^	16 (64.0%)	8 (66.7%)	8 (61.5%)	0.790
	(*N*=25)	(*N*=12)	(*N*=13)	
Osteosclerosis	98 (67.1%)	52 (71.2%)	46 (63.0%)	0.290
				
*Extravascular overload*				
** **Edema	121 (82.9%)	66 (90.4%)	55 (75.3%)	0.016
Ascites	54 (37.0%)	38 (52.1%)	16 (21.9%)	<0.001
Pleural effusion	45 (30.8%)	30 (41.1%)	15 (20.5%)	0.007
Pericardial effusion	81 (58.7%) (*N*=138)	44 (63.8%) (*N*=69)	37 (53.6%) (*N*=69)	0.226
Pulmonary hypertension	17 (12.3%)	13 (18.8%)	4 (5.8%)	0.020
	(*N*=138)	(*N*=69)	(*N*=69)	
Papilledema	73 (50.0%)	47 (64.4%)	26 (35.6%)	0.001
				
*Initial therapy*				
ASCT	62 (42.5%)	26 (35.6%)	36 (49.3%)	0.271
Melphalan-based therapy	6 (4.1%)	4 (5.5%)	2 (2.7%)	
Novel agents	77 (52.7%)	42 (57.5%)	35 (47.9%)	
Others^c^	1 (0.7%)	1 (1.4%)	0 (0.0%)	

a Comparisons between patients with higher and lower levels of serum β-CTX at baseline.

b Castleman's disease was diagnosed in 16 of 25 patients who underwent lymph node biopsies.

c Novel-agent group should include IMiDs (thalidomide and lenalidomide) and PIs (bortezomib); the others group included those patients using conventional chemotherapy (that is, CHOP).
