# Supplementary material for: Measurement of β-isomerized C-terminal telopeptide of type I collagen in patients with POEMS syndrome: diagnostic, prognostic, and follow-up utilities
Source: Blood Cancer J. 2016 Nov 11;6(11):e495–. doi: 10.1038/bcj.2016.109 (PMC5148056; doi:10.1038/bcj.2016.109)
Supplement: Supplementary Information [file bcj2016109x1.docx]

**Online Supplement material**

**Materials and Methods**

***Patients***

This study included 146 POEMS patients who were diagnosed and treated at Peking Union Medical College Hospital (Beijing, China) between January 2011 and March 2016. All of the patients met the diagnostic criteria proposed by Dispenzieri ^1^, as follows: (i) presence of both polyneuropathy and monoclonal gammopathy; (ii) presence of one of three major criteria (Castleman’s disease, osteosclerosis, or elevated VEGF); and (iii) one of six minor criteria (organomegaly, extravascular volume overload, endocrinopathy, skin changes, papilledema, and either thrombocytosis or polycythemia). All the patients have informed consent and this study has been approved by ethic committee of local hospital.

Detailed clinical features and laboratory information were collected at the time of diagnosis, as described previously ^2, 3^. Briefly, the overall neuropathy limitation scale (ONLS) was used to assess neurologic disability ^4^. Systolic pulmonary arterial pressure (sPAP) was estimated based on duplex cardiac ultrasonography, and pulmonary hypertension was defined as a sPAP ≥ 50 mmHg ^5^. Osteosclerotic bone lesions were detected by the bone window of chest/abdomen/pelvis computed tomography ^6^. Laboratory studies included serum protein electrophoresis, serum and urine immunofixation electrophoresis, and bone marrow examinations. The estimated glomerular filtration rate (eGFR) was calculated using the Chronic Kidney Disease Epidemiology Collaboration (CKD-EPI) equation ^7^.

Primary therapies included melphalan-based chemotherapy (N = 6), autologous stem cell transplantation (N = 62), novel agent-based therapy (N = 77), and others (i.e., CHOP chemotherapy, N = 1). The median time of follow-up was 24.2 months (range, 2.9–62.9 months). Clinical relapse was deﬁned as recurrence or deterioration of clinical symptoms attributable to POEMS syndrome after a complete or partial response ^8^.

***Serum PINP, β-CTX and VEGF measurements***

All the serums were obtained from fasting subjects in the early morning. Serum PINP and β-CTX levels were measured using a computer-controlled automatic analyzer (Roche Cobas E601; Holliston, MA, USA) for a chemiluminescence workstation with the use of Elecsys reagent kits (Roche Diagnostics, Basel, Switzerland). The normal ranges were 0.260–0.512 ng/ml and 0.268–0.440 ng/ml for males and females, respectively ^9^. Healthy volunteers (N = 23) and patients with disease manifestations similar to patients with POEMS syndrome, including chronic inflammatory demyelinating polyradiculoneuropathy (CIDP; N = 18), systemic lupus erythematosus (SLE; N = 35), multiple myeloma (MM; N = 13), and Langerhans’cell histiocytosis (LCH; N = 24), were used as normal and disease controls, respectively. Serum VEGF was measured with a human Quantikine ELISA Kit (normal < 600 pg/ml; R&D Systems, Minneapolis, MN, USA) ^10^. These two serum markers were measured simultaneously at the time of diagnosis, and after therapy (every 6 months-1 year).

***β-CTX, VEGF, and hematologic responses***

Complete response for serum markers was the normalization of levels after treatment (β-CTX less than gender-specific upper limit of normal; VEGF < 600 pg/ml). Hematologic complete response was deﬁned as the disappearance of monoclonal protein in serum and urine specimens ^3, 8^. No other levels of response were speciﬁed.

***Statistical analyses***

Analyses were performed with SPSS 22 (SPSS, Inc., Chicago, IL, USA). The chi-square test (Fisher exact test, when appropriate) and the Kruskal–Wallis method were used to ascertain differences between categorical and continuous variables, respectively. The relationship between continuous variables was detected using Spearman’s correlation analysis.

Overall survival (OS) and progression-free survival (PFS) were calculated from the date of treatment. For PFS analyses, death or progression were considered as events. Survival curves were plotted with the Kaplan–Meier method and compared with a log-rank test. All data were considered statistically signiﬁcant at a *p* < 0.05.

**References**

[1] Dispenzieri A. POEMS Syndrome: update on diagnosis, risk-stratification, and management. *Am J Hematol* 2015; 90(10):951-62.

[2] Li J, Zhou DB. New advances in the diagnosis and treatment of POEMS syndrome. *Br J Haematol* 2013; 161:303–15.

[3] Li J, Zhang W, Jiao L, Duan MH, Guan HZ, Zhu WG, et al. Combination of melphalan and dexamethasone for patients with newly diagnosed POEMS syndrome. *Blood* 2011; 117(24):6445-9.

[4] Graham RC, Hughes RA. A modified peripheral neuropathy scale: the Overall Neuropathy Limitations Scale. *J Neurol Neurosurg Psychiatry* 2006; 77:973–6.

[5] Li J, Tian Z, Zheng HY, Zhang W, Duan MH, Liu YT, et al. Pulmonary hypertension in POEMS syndrome. *Haematologica* 2013; 98(3):393-8.

[6] Glazebrook K, Guerra Bonilla FL, Johnson A, Leng S, Dispenzieri A. Computed tomography assessment of bone lesions in patients with POEMS syndrome. *Eur Radiol* 2015; 25(2):497-504.

[7] Levey AS, Stevens LA, Schmid CH, Zhang YL, Castro AF 3rd, Feldman HI, et al. A new equation to estimate glomerular filtration rate. *Ann Intern Med* 2009; 150: 604–612

[8] Cai QQ, Wang C, Cao XX, Cai H, Zhou DB, Li J, et al. Efficacy and safety of low dose lenalidomide plus dexamethasone in patients with relapsed or refractory POEMS syndrome. *Eur J Haematol* 2015; 95:325–30.

[9] Li M, Zhang ZL, Li Y, Deng WM, Deng ZL, LU F, et al. Re-analysis of serum procollagen type 1 N-terminal propeptide and β cross-linked C-telopeptide of type I collagen concentrations in healthy men and women of Han nationality. *Chinese Journal of Osteoporosis and Bone Mineral Research* 2016; (01) 7-13

[10] Wang C, Zhou YL, Cai H, Cheng XQ, Zhang W, Kang WY, et al. Markedly elevated serum total N-terminal propeptide of type I collagen is a novel marker for the diagnosis and follow-up of patients with POEMS syndrome. *Haematologica* 2014; 99:e78–80.

***Figure legends***

**Online Supplementary Figure 1.** Correlation between serum levels of β-CTX and PINP in POEMS patients (n=43).

**Online Supplementary Figure 2.** Overall survival and risk of progression in POEMS patients with and without (A) VEGF and (B) hematological normalization of therapies.
