# Supplementary material for: Measurement of β-isomerized C-terminal telopeptide of type I collagen in patients with POEMS syndrome: diagnostic, prognostic, and follow-up utilities
Source: Blood Cancer J. 2016 Nov 11;6(11):e495–. doi: 10.1038/bcj.2016.109 (PMC5148056; doi:10.1038/bcj.2016.109)
Supplement: Supplementary Table [file bcj2016109x2.doc]

**Table. Serial levels of serum VEGF and β-CTX in patients with relapsed disease course**

| **Pt No.** | **Cytokine** | **Diagnosis** | **1^st^ Remission** | **Relapse** | **2^nd^ Remission** | **Initial therapy** | **Remission period**  **(months)** | **Salvage therapy** |
| --- | --- | --- | --- | --- | --- | --- | --- | --- |
| 1 | VEGF | 2439 | 174 | 1943 | 63 | ASCT | 36 | LDex |
|  | β-CTX | 0.9 | 0.4 | 0.9 | 0.494 |  |  |  |
| 2 | VEGF | 5980 | 2859 | 4232 |  | ASCT | 24 | LDex |
|  | β-CTX | 1.1 | 0.6 | 0.789 |  |  |  |  |
| 3 | VEGF | 5668 | 729 | 6535 |  | ASCT | 27 | LDex |
|  | β-CTX | 1.7 | 1 | 1.29 |  |  |  |  |
| 4 | VEGF | 2890 | 98 | 1876 |  | ASCT | 29 | LDex |
|  | β-CTX | 0.8 | 0.7 | 1.48 |  |  |  |  |
| 5 | VEGF | 8052 | 543 | 5824 |  | LDex | 12 | Follow-up |
|  | β-CTX | 1.3 | 0.6 | 1.030 |  |  |  |  |

Pt. No., patient number; VEGF, vascular endothelial growth factor; β-CTX, β-isomerized C-terminal telopeptide; ASCT, autologous stem cell transplantation; LDex, lenalidomide and dexamethasone
