# Supplementary figures and images for: Measurement of β-isomerized C-terminal telopeptide of type I collagen in patients with POEMS syndrome: diagnostic, prognostic, and follow-up utilities
Source: Blood Cancer J. 2016 Nov 11;6(11):e495–. doi: 10.1038/bcj.2016.109 (PMC5148056; doi:10.1038/bcj.2016.109)

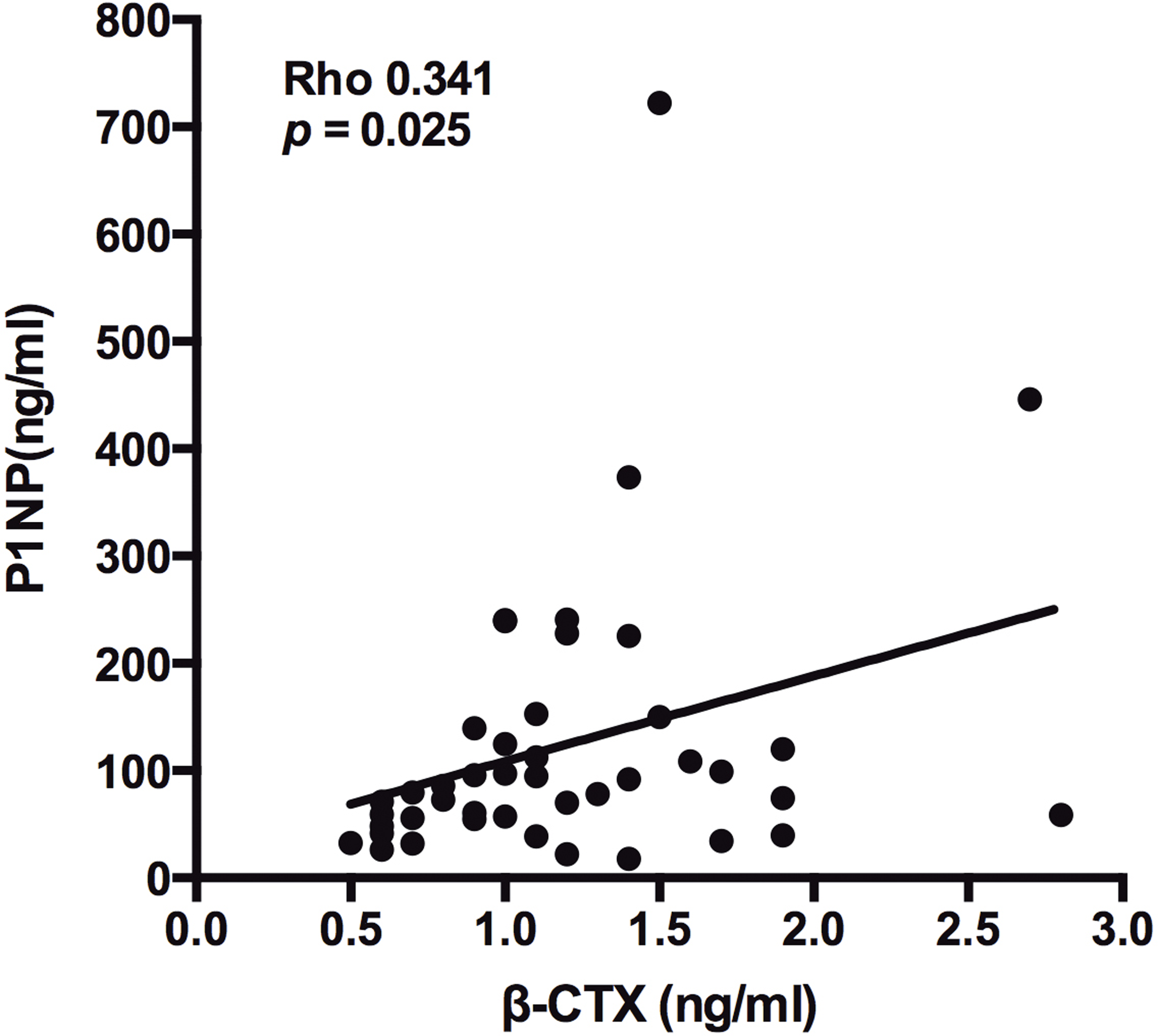

Supplement: Supplementary Figure 1 [file bcj2016109x3.tif]

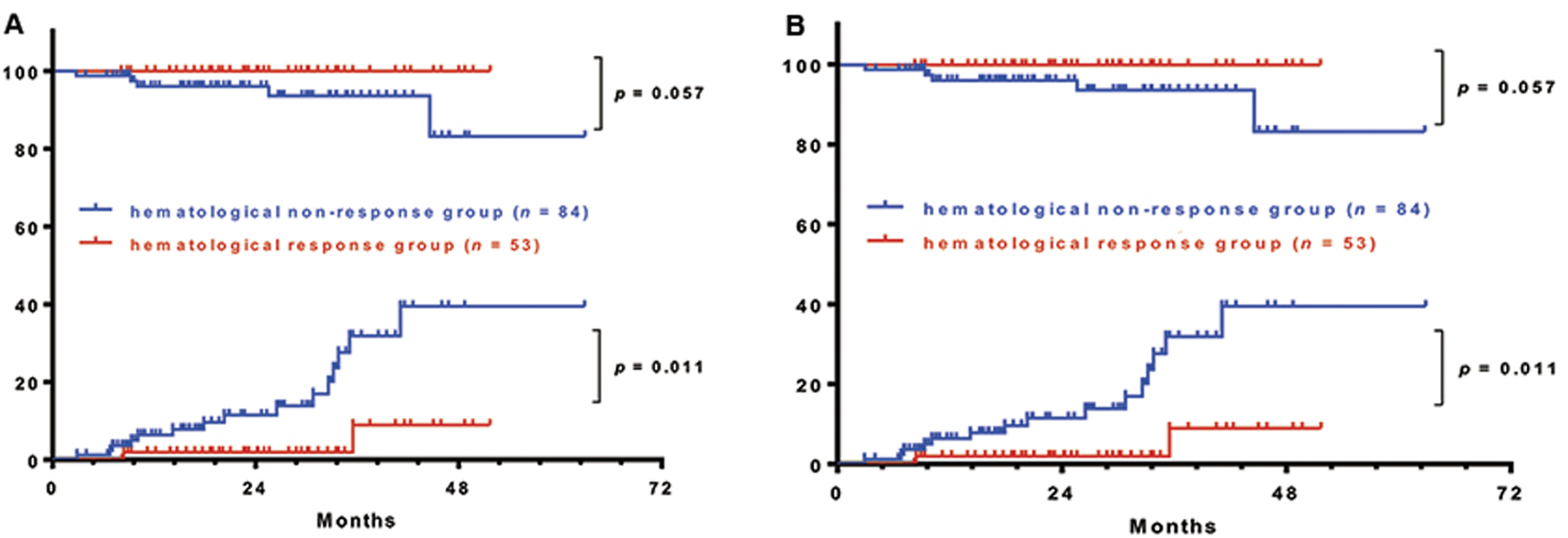

Supplement: Supplementary Figure 2 [file bcj2016109x4.tif]
